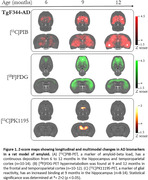# Longitudinal changes in amyloid, glucose, and glial reactivity imaging biomarkers in a transgenic rat model of amyloidosis

**DOI:** 10.1002/alz.092884

**Published:** 2025-01-09

**Authors:** Gabriela Lazzarotto, Lidia Emmanuela Wiazowski Spelta, Cleinando Clemente da Silva Vera, Andreia Silva da Rocha, Débora Guerini de Souza, Daniele de Paula de Paula Faria, Eduardo R. Zimmer

**Affiliations:** ^1^ UFRGS, Porto Alegre Brazil; ^2^ USP, São Paulo Brazil; ^3^ University of Pittsburgh, Pittsburgh, PA USA; ^4^ Universidade Federal do Rio Grande do Sul, Porto Alegre, RS Brazil

## Abstract

**Background:**

Imaging biomarkers have helped to reconceptualize Alzheimer's disease pathophysiology. More specifically, positron emission tomography (PET) radiopharmaceuticals for non‐invasively assessing amyloid‐beta (Aβ) plaques, glucose metabolism, and glial reactivity allow for tracking disease progression in a temporally ordered manner. However, whether transgenic models recapitulate biomarker‐related changes remains elusive. Here, we longitudinally evaluated Aβ, glucose metabolism, and glial reactivity using PET in a transgenic amyloid rat model.

**Method:**

TGF344‐AD (APP/PS1) and wild‐type rats were scanned at 6, 9, and 12 months with [^18^F]FDG, [^11^C]PK11195 and [^11^C]PIB. Images were manually co‐registered in a rat magnetic resonance template. Standardized uptake values (SUV) were calculated for [^18^F]FDG and [^11^C]PK11195, and SUV ratio (SUVR) was calculated for [^11^C]PIB using the pons as the reference region. The results were normalized using Z‐Score and statistical significance was determined at Z>2 (p < 0.05).

**Result:**

At 6 months, no differences between groups were observed. At 9 months, however, we detected significant amyloid load, glucose hypermetabolism, and increased glial reactivity. At 12 months, animals presented increased Aβ load while still presenting signs of brain glucose hypermetabolism. By contrast, glial reactivity did not present alterations compared to 9‐month‐old rats.

**Conclusion:**

Our findings indicate that in the early phases of detectable Aβ deposition, there is a joint increase in brain glucose metabolism and glial reactivity. These findings suggest that amyloid deposition triggers early neuroinflammatory response. We and others have provided evidence that early FDG‐PET hypermetabolism is an indicator of glial reactivity.